# Evaluating the effect of VersaWrap tendon protector on functional outcomes in operative tendon repairs

**DOI:** 10.3389/fsurg.2024.1447515

**Published:** 2024-12-16

**Authors:** Yaw Adu, Justin Harder, Cameron Cox, Gracie Baum, Evan J. Hernandez, Brendan J. MacKay

**Affiliations:** Department of Orthopaedic Hand Surgery, Texas Tech University Health Sciences Center, Lubbock, TX, United States

**Keywords:** tendon, operative tendon repairs, tendon protector, bioresorbable hydrogel sheet, VersaWrap

## Abstract

**Background:**

Tendon repairs often result in adhesion formation which can cause persisting functional deficits. Close proximity of healing tissues increases friction during tendon excursion, often leading to tendon tethering postoperatively. Despite continued improvements in techniques for tendon repairs, there is currently no consensus on the most effective modality to reduce adhesion formation. The VersaWrap Tendon Protector is a bioresorbable hydrogel that is FDA-cleared for use in tendon repair by separating healing tendons from surrounding tissues and improving tendon gliding. We conducted a study to assess the efficacy of VersaWrap in improving clinical outcomes related to adhesions and tethering in tendon repairs involving the hand.

**Materials & methods:**

Age, sex, injury type, mechanism of injury, visual analogue scale (VAS) pain scores, active and passive range of motion (ROM), percent return to normal function, and patient-reported outcomes forms (QuickDASH) were collected at baseline and routine follow up visits. Functional outcomes were classified according to Strickland and Glogovac grading system.

**Results:**

90 patients were included, with an average age of 39.8 years and a 44% female gender. The most common mechanism of injury was sharp laceration, and the majority of repairs involved the extensor mechanism (58.8% extensor, 35.3% flexor, 5.8% both). At a mean follow-up of 4.6 months, the mean active and passive ROM was 88.8% and 94.3%, respectively. Mean percent return of function was 87.7%. Good or Excellent functional outcomes were achieved in 92.3% of patients – 70.5% Excellent, 21.8% Good, 6.4% Fair, 1.3% Poor. The average QuickDASH score was 30.7, and the average final VAS pain score was 1.3.

**Conclusions:**

Tendon repairs and tenolysis procedures often result in reduced functionality due to impeded tendon gliding, and there is currently no consensus on optimal treatment to prevent tethering to surrounding tissues. The VersaWrap Tendon Protector creates a gelatinous layer between the tendon and surrounding soft tissue to improve gliding resistance, thereby limiting tendon sheath adhesions. Our data suggests that VersaWrap may be a useful adjunct in preventing tendon tethering adhesion post-repair.

## 1 Introduction

The hands are frequently involved in traumatic and/or chronic injuries, representing up to 30% of presentations to urgent or emergency care centers (1). Tendon injuries are the second most common injury of the hand and can result in long-term disability if not treated appropriately (2, 3). These injuries are thought to be caused by either mechanical (excess loading on tendon) or vascular (loss of blood supply leading to ischemic damage) processes, with athletes and individuals in high-risk occupations such as construction being at a higher risk (4, 5). These injuries can manifest in various forms, such as tendinopathies, tendinosis, and tendinitis, and as such, various treatment methods have been developed to restore patient function (6, 7). The initial approach to treating tendon injuries is typically conservative and includes rest, physical therapy, and medication (2). However, even non-steroidal anti-inflammatory drugs (NSAIDS) and steroids, which are commonly used to relieve pain and inflammation, have shown negative effects on tendon healing in the long term, and are typically used as last resort conservative treatments (4, 8, 9). Alternative treatments, such as transdermal nitric oxide patches, shock wave therapy, and eccentric loading exercises, have also shown promise in treating tendon injuries in which there is not complete rupture or transection (4, 10, 11). In cases where conservative treatment is unsuccessful and in more severe traumatic injuries, surgical intervention is often indicated.

Surgical approaches vary depending on the severity and location of the injury (2). The primary goal of surgery is to achieve intrinsic tendon healing and limit extrinsic scarring, with the ultimate goal of maximizing patient functional outcomes (12). Operative interventions, while often successful, can lead to a high rate of reoperations, with one report showing a 11.4% reoperation rate following flexor tendon repair (12, 13). Surgical procedures may also present with a risk of complications post operation, such as tendon rupture, joint stiffness, poor tendon gliding, and adhesion formation (14, 15).

Adhesion formation and excess scarring are of particular concern, as impairments to gliding and anatomic motion can progress to permanent deficits or the need for reoperation (12, 13). Adhesions can arise from a variety of tissues surrounding the repair, such as dense adhesions arising from the bony floor, loose adhesions arising from subcutaneous tissues, or moderate adhesions arising from the synovial sheath or pulleys (15, 16). It is estimated that adhesions develop in 30% of tendon repairs, and while modified suture techniques such as the Kessler repair method and early mobilization have improved adhesion-related functional deficits to some degree, outcomes of flexor and extensor tendon repairs of the hand remain suboptimal (3, 17, 18).

Recently, novel uses of tissue barriers and pharmacologic agents (both alone and in combination) have been explored as potential adjuncts to adhesions in tendon repair, including hyaluronic acid (HA) preparations, lubricin (also known as proteoglycan 4), di-palmitoyl phosphatidylcholine, 5-flourouracil, celoxumib sheets, and acellular dermal matrix products (19, 20). While some of these have shown potential for reducing adhesions and scarring, the majority of studies have been performed on animal models or *in vitro*, and their clinical utility in the context of tendon repair has yet to be established (19).

The VersaWrap tendon protector (Alafair BioSciences, Austin, TX, USA) is a plant-based (non-tissue, non-collagenous) bioresorbable hydrogel sheet that was developed to protect tendons, ligaments, skeletal muscle, and peripheral nerve. VersaWrap provides an interface between tissues that reduces friction and prevents tethering (21). In the context of tendon repairs in the hand, we postulated that this product may be a useful adjunct to prevent adhesion formation and scarring in healing tendons, with the ultimate goal of improved tendon gliding. Given the promising early data of VersaWrap as a tendon protector, we designed a study to evaluate the clinical efficacy of VersaWrap in tendon repairs of the hand. Given the unique function of this device in reducing adhesion formation and friction, we hypothesized that tendon repairs utilizing this novel tissue barrier would result in improved functional outcomes.

## 2 Methods

We performed a retrospective study of patients who underwent surgeries using the VersaWrap tendon protector over a period of 2 years. In all cases, a 2 × 2 inch sheet preparation of VersaWrap (VTP 2201) was cut to the appropriate length and wrapped around the affected zone circumferentially immediately following repair. No adjustments in repair technique were made to facilitate the use of VersaWrap. All procedures were performed by two fellowship trained hand surgeons, each with over 10 years of experience.

Demographic information, injury characteristics, and functional measures were collected, including: age, sex, tendons injured, mechanism of injury, pain scores, disability scores, range of motion, and percent return to normal function. These variables were assessed at baseline and at routine follow-up visits until patients had reached maximal recovery or were lost to follow up. Functional outcomes were further categorized using the Strickland and Glogovac grading system, and disability scores were calculated at final follow up using the Shortened Disabilities of the Arm, Shoulder, and Hand (QuickDASH) questionnaire when possible (Tables 1–4) (2, 22). Descriptive statistics were used to evaluate clinical endpoints.

**Table 1:** Strickland and glogovac grading criteria.

Grade	Total active range of motion (degrees)	Functional return (%)
Excellent	>150°	85–100
Good	125°–149°	70–84
Fair	90°–124°	50–60
Poor	<90°	0–49

**Table 2:** Cohort demographic and injury characteristics.

Age	39.8 years
Sex	44% Female
Tendon involved	Extensor (58.82%)
	Flexor (35.29%)
	Both (5.82%)
Mechanism of injury	Traumatic laceration (31.67%)
	Traumatic fracture (11.67%)
	De Quervain's tenosynovitis (10.83%)
	Chronic wrist pain (9.17%)

**Table 3:** Outcomes of tendon repairs using VersaWrap.

Strickland and Glogovac grade	Excellent	Good	Poor	Fair
All flexor tendon repairs (*n* = 34)	67.6% (23)	23.5% (8)	5.9% (2)	2.9% (1)
Flexor tendon only (*n* = 23)	69.6% (16)	21.7% (5)	8.7% (2)	0.0%
All extensor tendon repairs (*n* = 41)	78.0% (32)	22.0% (9)	0.0%	0.0%
Extensor tendon only (*n* = 30)	83.3% (25)	16.7% (5)	0.0%	0.0%
Concomitant flexor and extensor tendon repairs (*n* = 11)				
Flexor tendon	63.6% (7)	27.3% (3)	0.0%	9.1% (1)
Extensor tendon	63.6% (7)	36.4% (4)	0.0%	0.0%
All repairs (*n* = 78)	70.5%	21.8%	6.4%	1.3%
QuickDASH score	Mean ± St. Dev. (Range)			
All flexor tendon repairs (*n* = 19)	31.6 ± 20.5 (2.3–70.8)			
Flexor tendon only (*n* = 12)	29.4 ± 20.8 (2.3–63.6)			
All extensor tendon repairs (*n* = 25)	29.9 ± 24.1 (0.0–72.5)			
Extensor tendon only (*n* = 18)	31.4 ± 23.0 (0.0–72.5)			
Concomitant flexor and extensor tendon repairs (*n* = 7)	40.8 ± 25.8			
All repairs (*n* = 44)	30.7 ± 22.0 (0.0–72.5)			

**Table 4:** VersaWrap tendon repair outcomes classified by zone of involvement.

Flexor tendons	Strickland grade			
	Excellent	Good	Fair	Poor
Zone 1 (*n* = 5)	60.0%	20.0%	0.0%	20.0%
Zone 2 (*n* = 19)	57.9%	31.6%	10.5%	0.0%
Zone 3 (*n* = 3)	66.7%	33.3%	0.0%	0.0%
Zone 4 (*n* = 7)	85.7%	0.0%	14.3%	0.0%
Zone 5 (*n* = 1)	0.0%	100.0%	0.0%	0.0%
T1 (*n* = 0)	–	–	–	–
T2 (*n* = 1)	100.0%	0.0%	0.0%	0.0%
T3 (*n* = 2)	100.0%	0.0%	0.0%	0.0%
Extensor tendons	Strickland grade			
	Excellent	Good	Fair	Poor
Zone 1 (*n* = 2)	50.0%	0.0%	50.0%	0.0%
Zone 2 (*n* = 5)	60.0%	0.0%	40.0%	0.0%
Zone 3 (*n* = 5)	60.0%	20.0%	20.0%	0.0%
Zone 4 (*n* = 4)	50.0%	25.0%	25.0%	0.0%
Zone 5 (*n* = 13)	69.2%	23.1%	7.7%	0.0%
Zone 6 (*n* = 11)	81.8%	9.1%	9.1%	0.0%
Zone 7 (*n* = 6)	66.7%	16.7%	16.7%	0.0%
Zone 8 (*n* = 3)	66.7%	33.3%	0.0%	0.0%

## 3 Results

In the specified time frame, 120 patients had tenolysis and/or tendon reconstruction with the use of a VersaWrap tendon protector. Patients with follow up <1 month were excluded from analysis. A total of 90 patients were included in the analysis, with an average age of 39.8 years and a 44% female gender. The most common mechanism of injury was sharp laceration, and the majority of repairs involved the extensor mechanism (58.8% extensor, 35.3% flexor, 5.8% both flexor and extensor repairs) (Table 2).

At most recent follow-up (mean: 4.6 months, range: 1.0–19.3), the mean active and passive ROM were 88.8% (*n* = 66, range: 40%–100%) and 94.3% (*n* = 53, range: 50%–100%), respectively. The average percent return of function was 87.7% (*n* = 59, range: 30%–100%). Classification using Strickland and Glogovac criteria showed 92.3% of patients had Good or Excellent functional outcomes (70.5% Excellent, 21.8% Good, 6.4% Fair, 1.3% Poor) (Table 3). The average QuickDASH score was 30.7 (*n* = 44, range: 0–72.5), and the average pain score on the visual analogue scale (VAS) was 1.3/10 (*n* = 90, range: 0–8) (Table 3). Outcomes of flexor and extensor tendon repairs classified by zone of involvement can be found in Table 4.

## 4 Discussion

After tendon injury and/or repair, healing is achieved via extrinsic and intrinsic processes. Extrinsic healing involves a distinctive inflammatory response followed by proliferation and remodeling, in which fibroblasts of the paratenon facilitate migration and may result in adhesion formation, particularly with prolonged immobilization. The intrinsic process is supported by tendon movement and is carried out by “fibroblast-like tenocytes” which produce collagenous tissue and drive remodeling (1). Minimizing the inflammatory response is critical to preventing adhesions and/or excessive scarring surrounding the tendon repair, and early motion is critical to healing and remodeling of the tendon itself. Close proximity of hand tendons to surrounding structures creates an environment favorable for adhesions during the inflammatory stage of healing (1). These adhesions can lead to restricted tendon movement, and once tendon adhesion has developed, it is unlikely that the patient will achieve excellent range of motion.

Recent clinical studies on 315 primary flexor tendon repairs reported that 28% of flexor tendon repairs had only a fair to poor functional recovery, likely as a result of adhesion formation (23). One meta-analysis consolidated 20 major reports over the course of 15 years to track outcomes of flexor tendon repairs in the hand. In this report, Tang et al. found that 4%–10% of repairs ruptured, and 10% developed restrictive adhesion leading to tenolysis or secondary grafting (15).

In a study of 225 primary flexor tendon repairs, “Excellent” or “Good” function was achieved by only 30% of patients at 8 weeks and 48% of patients at final follow-up (minimum 3 months). A systematic review of flexor tendon repairs found rates of “Excellent” or “Good” functional recovery varied from 61%–82% in adult patients (24, 25). In our cohort, 87% of patients achieved “Excellent” or “Good” functional status after flexor tendon repair with the VersaWrap tendon protector (Table 3).

Extensor tendon repairs are often analyzed separately from flexor tendons due to differences in anatomy and their potential effects on prognosis (1). One study reported only 31% improvement in total active motion from tenolysis performed after extensor tendon repair (26). In a study of 101 extensor tendon injuries, Newport et al. reported “Excellent” or “Good” results in 50% of repairs in zones 1–4 and 63%–83% of repairs in zones 5–8 (27). A more recent study of 72 extensor tendon repairs showed “Excellent” or “Good” outcomes in 75% of patients (42% Excellent, 33% Good, 13% Fair, 13% Poor) (28). While attempts have been made to establish prognosis by zone of injury involved, the current literature is conflicted with regard to which zones are likely to have better or worse outcomes (29). In our cohort, 91% of patients achieved “Excellent” or “Good” functional status after extensor tendon repair with the VersaWrap tendon protector (Table 3).

The majority of studies evaluating both flexor and extensor tendon repairs have excluded patients with concomitant injuries such as fractures, complex skin injuries, or revascularisations (23–25, 27–29). Despite including all patients with adequate follow-up, regardless of concomitant injuries or complex reconstruction, our results compare favorably to the historical outcomes of both flexor and extensor tendons of the hand. These findings indicate that the VersaWrap tendon protector may be a valuable adjunct in tendon repairs resulting from a wide variety of injuries.

The present study is not without limitations. Injuries in our cohort varied in terms of mechanism and chronicity of injury. While aggregate outcomes are promising in our cohort, comparative analysis between zones of injury is limited by sample size (Tables 3, 4). Further, there was no control group to provide definitive comparison of VersaWrap vs. non-VersaWrap repairs. Despite these limitations, our study provides valuable data regarding the efficacy of this novel product in improving gliding and preventing complications related to scarring and/or adhesions.

## 5 Conclusion

Tendon injuries and tendinopathies are a prevalent issue that affects individuals of all ages and backgrounds and can greatly impact a patient's functionality. Functional recovery is often dependent on the degree of scarring and adhesions post-repair. The gelatinous layer provided by VersaWrap between the tendon and surrounding soft tissue reduces friction and improves gliding, thereby limiting the formation of tendon sheath adhesions. Our data suggests that the VersaWrap Tendon Protector may be a useful adjunct in preventing tendon adhesion and excessive scarring, ultimately leading to improved outcomes in patients undergoing tendon repairs of the hand.

## Data Availability

The raw data supporting the conclusions of this article will be made available by the authors, without undue reservation.
